# Resident Well-Being and Staff Practices in Dementia Villages: Insights From Two Case Studies

**DOI:** 10.1093/geroni/igaf122.1151

**Published:** 2025-12-31

**Authors:** Habib Chaudhury, Ziying Zhang, Myia Wilhelm

**Affiliations:** Simon Fraser University, Burnaby, British Columbia, Canada; Simon Fraser University, Burnaby, British Columbia, Canada; Simon Fraser University, Vancouver, British Columbia, Canada

## Abstract

Over the last decade, “Dementia Villages” have emerged as an innovative model of long-term care, emphasizing small households and residents’ access to amenities and destinations within a community. This study examines the village model’s impact through two case studies in British Columbia, Canada: The Views Village and The Village Langley. Using a multi-method approach, we conducted standardized environmental assessments and staff interviews to explore how these environments influence residents’ quality of life and staff’s care practices. In each village, interviews were conducted with administrative and frontline staff members. Our findings show that the village model fosters resident autonomy and engagement, while requiring staff adaptation to new care approaches. For residents, the community environment offers a variety of activities and destinations, providing greater choice and flexibility in daily engagement. The small household model enhances staff-resident communication, allowing for personalized care. Environmental features like open kitchens, private bedrooms, and homelike décor promote autonomy and a sense of ownership. For staff, interviews revealed both opportunities and challenges associated with the village model. Staff experienced a transition from task-oriented to person-centered care and witnessed the significant benefits of the model for the residents. The new care model introduced higher skill requirements for frontline staff and emphasizes interdisciplinary collaboration, which requires a significant cultural shift and demands adaptation from care staff. This study contributes to the development of evidence-base in the dementia village model, offering practical insights into how innovative environments and care approaches may enhance resident well-being and staff experiences in long-term care settings.

